# Temporal and spatial distribution of under-five mortality and factors associated with multiple cases of under-five deaths within a family in the rural area of Khuzestan, Southern Iran

**DOI:** 10.1038/s41598-018-36438-5

**Published:** 2018-12-18

**Authors:** Tofigh Anafcheh, Mahmoud Yaghoubi Doust, Mehdi Mojadam, Roksana Mirkazemi, Morteza Abdullatif Khafaie

**Affiliations:** 1 Department of Public Health, Faculty of Health, Ahvaz Jundishapur University of Medical Sciences, Ahvaz, Iran; 2 Department of Sociology, Payame Noor University, Kianpars Ave, Ahvaz, Iran; 3 Research and Development Institute of Farzanegan-e NikAndish, No.16, Abazar Blvd, Kashani Blvd, Tehran, Iran; 4 Social Determinants of Health Research Center, Ahvaz Jundishapur University of Medical Sciences, Ahvaz, Iran

## Abstract

Under-five mortality (U5M) is an important indicator of the overall health and development of society. There is a wide gap in U5M among different countries and also within the countries. This study was carried out to assess the prevalence, as well as the socio-demographic, and health-related causes of U5M in the region of study. A cross-sectional study was conducted among all registered cases of U5M in rural areas of Khuzestan province, Iran, during the years 2011 to 2015. To assess the socio-demographic determinants of U5M, the sample surveyed consists of 320 families with at least one under-five death using a multistage random sampling method. Also, this study evaluated the number of variables, which may increase the chance of families to have more than one U5M. U5M was 26 per 1000 live births in 2011, but decreased to 22 per 1000 live births in 2015. With the highest cumulative incidence of 43 in Masjed Soleyman and the lowest of 15 in Dehdez, infant mortality constitutes 76% of all U5M. Prematurity and congenital anomalies were responsible for 46% of all causes of mortality (that is, U5). Maternal age at delivery <18 years or >35 years (OR = 3.5; 95% CI, 1.29–6.22), marriage duration >9 years (1.85, 1.06–3.21), spouse age gap >5 years (2.32, 1.20–4.50), cesarean section (3.85, 1.19–5.74), delivery interval <3 years (2.83, 1.22–5.58), non-Arab ethnicity (2.58, 1.50–4.44), and mother working in agriculture or animal husbandry (3.93, 1.41–6.94) were the most important determinants associated with more than one child death per family. Spatially, there was a great disparity in U5M with different reduction rate during the 5 years of the study. Marriage age, cesarean delivery, delivery interval, and mother field activity were associated with U5M. This may have implications for the preventive health program.

## Introduction

Under-five mortality (U5M), the risk of a child dying before completing the five first years of life, is one of the most important indicators of the overall health and development of societies^1^. Globally, 5.9 million under-five deaths occurred in 2015, which is in line with the 16000 cases each day^2^.

Recently, an accelerated decline in U5M was recorded^3^, with a huge gap in the annualized reduction rate which is a result of the endeavor of countries to achieve the Millennium Development Goal 4 (MDG-4) of reducing U5M to at least as low as 25 per 1000 live births^4^. However, this forced action for proceeding towards the MDG-4 in many countries has resulted in an increase in inequality and disparity among the privileged and underprivileged strata of society^5^.

There is a wide gap in U5M among not only different countries (developed and developing countries) but also within the countries^6^. Rural, poor, and marginalized societies, that are the neediest, are the least beneficial of health promotion activities and services.

Like many other countries, Iran witnessed a steady decline in U5M from 281 cases per 1000 live births in the year 1956 to 16.8 cases per 1000 live births in the year 2013^7^. However, a wide gap has been reported in U5M among rural and urban areas and among different provinces of Iran^8,9^.

Many factors influence U5M, among them, overall socio-economic^10^ and access to health care^11^ play a crucial role. Other determinants are parental educational level, especially mothers’ literacy level^10,12^, gender discrimination level especially mother decision making power^13^, residential area (urban vs. rural and poor neighborhood)^14^, and birth interval^15^.

Khuzestan is an oil and gas province located in Southwest Iran, bordering Iraq and the Persian Gulf. There are more than four different ethnic groups, mostly Arab and Bakhtiari tribes. This province was the most affected during the eight-year war between Iran and Iraq. Previous studies have shown a high level of disparity in U5M within the province as compared to other provinces^8,9^. Therefore, this study evaluated the prevalence, socio-demographic, and health-related causes of U5M in the rural areas of Khuzestan province, Iran.

## Methods

U5M information was obtained from the corresponding rural areas of 19 cities (out of 28) of Khuzestan province during the years 2011 to 2015. U5M data were derived from the death registry of Ahvaz Jundishapur University of Medical Sciences, Department of Health.

### Sampling

To assess the socio-demographic determinants and health-related information of a family with at least one case of under-five child death, 320 cases were selected as the sample by using the multistage random sampling method. First, all cases of U5M were stratified to the five-year mortality groups in rural areas of the 19 cities in Khuzestan province (19 × 5 = 95 strata), then from each stratum, samples were selected in proportion to the population (of U5M) of that stratum.

### Data collection

A data collection form was developed which elicited information such as weight at birth of the deceased child, history of abortion, access to health house (received at least 4 antenatal care), place of residence, whether households were residing in villages which performed “Hadi Plan” (also called “Tarh e Hadi” or physical guide plan that facilitated 14500 villages by providing extensive physical improvement to the areas such as change in the physical texture of the village, change in the pattern of housing construction, coordination of rural road networks, and facilitating the traffic of villagers)^16^, family income, occupation, education, ethnicity, insurance scheme, delivery interval (as an indicator variable; below 3 vs. above 3 years interval between the deceased child and the deliver prior to that child), the number of pregnancies, age of mother at the time of marriage, age of mother at first pregnancy, age of mother at the time of delivery of deceased child, the number of children, and history of U5M in previous pregnancies (in addition to the case in this study). The data collection forms was administered by a health house worker (Behvarz) to the 320 mothers with a case of U5M. This was done in accordance with the approved guidelines and informed consent was obtained from all the respondents. The study protocols and procedures were approved by the Ethical committee, Deputy of Research and Development, Ahvaz Jundishapur University of Medical Sciences (IR.AJUMS.REC.2016.213).

### Statistical analysis

For descriptive statistics, Chi-square test and analysis of variance (ANOVA) were used. ArcGIS software was used to map the cases of U5M along with the spatial distribution of the health house. Multiple logistic regression was used to determine the important factors related to having >1 U5M (dependent variable, families with more than one case of U5M = 1 vs. families with one case of U5M = 0). The variables used in the regression were first investigated for possible multicollinearity. The odds ratio (Exp. β, 95% CI) of each variable was estimated by determining its importance in the presence of more than one child death in families. The significance threshold of P = 0.05 was used in all the analyses. All statistical analyses were performed using STATA version 14.1 software (STATA Corporation, College Station, TX).

### Ethics approval

The study was approved by the Deputy of Research and Development, Ahvaz Jundishapur University of Medical Sciences (IR.AJUMS.REC.2016.213).

## Results

### Under-five incidence rate

**Figure 1:**
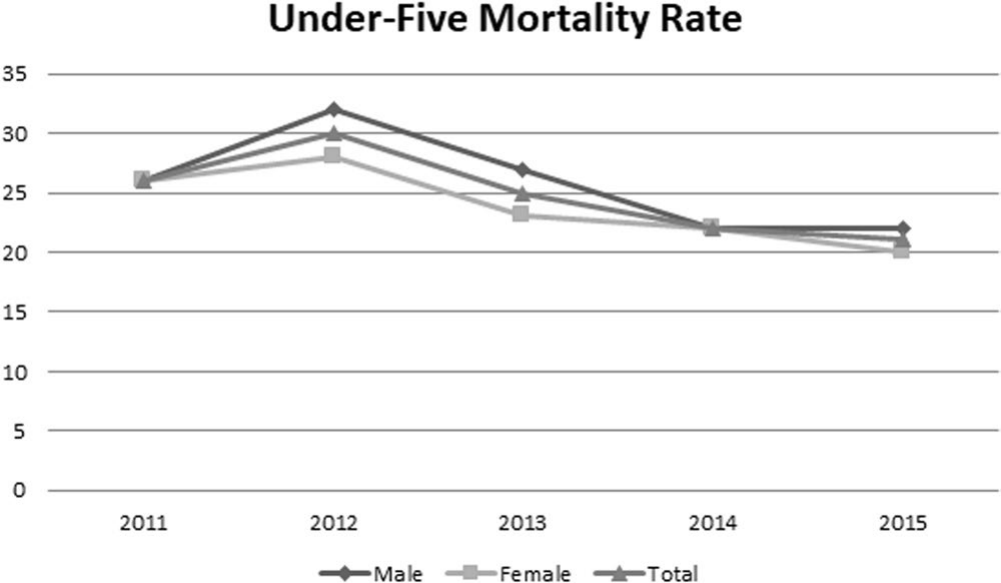
The annual under-five mortality rate between 2011 and 2015 in Khuzestan Province, Iran.

**Figure 2:**
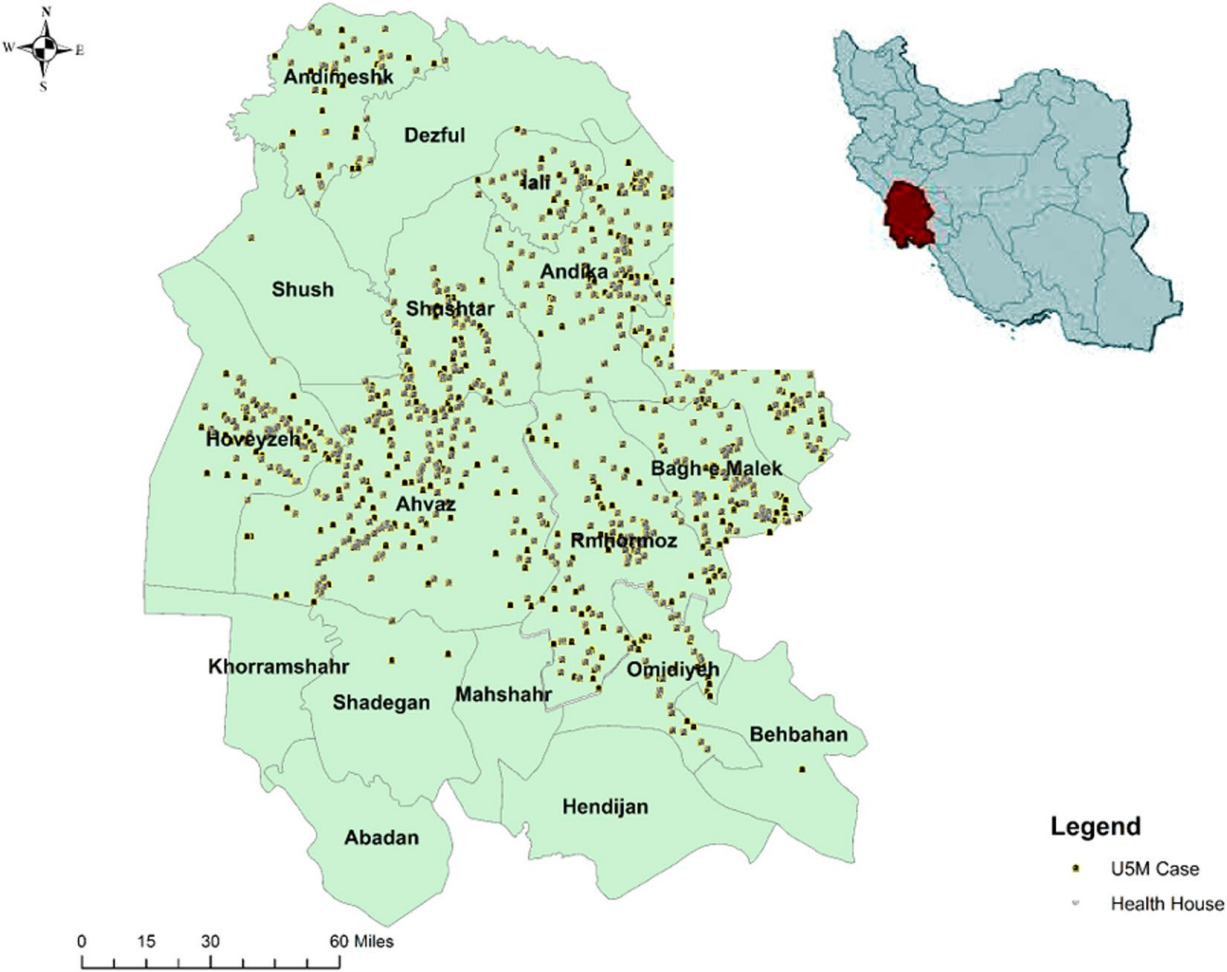
Spatial distribution of under-five mortality cases (n = 1973) and health house located in the rural area of Khuzestan province, Iran.

U5M rate between 2011 and 2015 is as shown in Fig. 1. Also, the spatial distribution of U5M is as shown in Fig. 2. There was a total of 1973 cases of U5M during the five years period. Under five mortalities in 2011 were 26 per 1000 live births, then a rise was observed in the next year (that is, U5M in 2012 = 30) which gradually reduced to 22 per 1000 live births in 2015. However, the different area experienced different scenario, for instances Andimeshk and Hamidiyeh observed higher U5M in 2015 than 2011 and Dehdez showed a fluctuating trend during the 5-year period (Table 1). There was a great disparity in the mortality rate between rural areas within the province. The maximum cumulative U5M was 43 per 1000 live births in five years in rural areas of Masjed Soleyman and a minimum of 15 per 1000 live births for rural areas of Dehdaz city with an average of 25 in all the areas. Age of mothers at first pregnancy was 21 ± 4.5 (ranging from 14 to 46 years). In summary, the male and female U5M rate ve were 25.8 and 23.8 per 1000 live births, respectively. Fifty-five percent of deaths occurred below one month (n = 1094) and 21.0% (n = 409) of deceased children were between one month and one year.

**Table 1:** The under-five mortality rate in Khuzestan province per 1000 live births by sex between 2011 and 2015.

County	2011		2012		2013		2014		2015		5 yrs. Cumulative Rate
	M	F	M	F	M	F	M	F	M	F	
Omidiyeh	38	22	32	26	13	18	13	14	10	8	19
Andimeshk	25	16	45	14	54	25	22	19	67	25	31
East Ahvaz	0	0	26	20	33	45	37	15	13	27	22
Bavi	14	5	11	18	30	46	31	22	10	11	20
Karoon	7	7	16	19	40	38	5	5	29	26	19
West Ahvaz	22	33	52	32	33	35	21	24	21	5	28
Hamidiyeh	13	15	20	12	15	15	10	28	36	35	20
Izeh	21	22	40	18	28	16	13	14	10	20	20
Bagh-e Malek	17	19	33	30	17	24	20	19	11	13	20
Dasht-e Azadegan	73	87	29	53	30	27	25	24	19	17	38
Dehdez	11	12	22	11	5	0	21	11	40	19	15
Ramshir	48	40	35	36	22	13	20	26	20	17	28
Ramhormoz	16	24	29	24	20	21	13	27	10	26	21
Shushtar	34	25	42	42	23	22	32	32	30	25	31
Lali	5	5	30	23	28	53	16	12	90	42	31
Masjed Soleyman	12	38	25	0	30	0	82	149	53	38	43
Andika	16	21	32	39	40	4	27	18	4	10	21
Haftkel	16	0	14	26	27	30	66	33	16	24	25
Hoveyzeh	47	46	22	33	28	9	30	19	8	39	28
Total	26	26	32	28	27	23	22	22	22	20	25

### Socio-demographic and health-related characteristics of families

Out of the 320 families, 280 subjects participated in the present survey (87.5% response rate). Among the subsample of families with U5M, a fair proportion of the total number of subject was recruited from central villages (with a Health House), satellite villages, and nomads without a permanent residence. The majority were Arab (59% of the participants). More than 60% of the subjects reside in a rural area where Hadi Plan was implemented. The literacy rate of male and female in this area were 88 and 84%, respectively. More than 94% of the mothers were housewives and 19% of the fathers were unemployed. Twenty-nine percent of the participants had a monthly income of <$60 USD (Table 1)^17^. The almost equal proportion of marriage was arranged or free choice and spouse age gap ranged from 0 to 20 years (Table 2).

**Table 2:** Socio-demographic characteristics of families with case of U5M in the previous five years in rural areas Khuzestan Province.

Characteristics	1 U5M n = 206	>1 U5M n = 74	Total n = 280
**Location**			
Central village	136 (66.0)	49 (66.2)	185 (66.1)
Satellite village	65 (31.5)	25 (33.8)	90 (32.1)
Nomad areas	5 (2.4)	0 (0.0)	5 (1.8)
**Ethnicity**			
Arab	134 (65.04)	31 (41.9)	165 (58.9)
Bakhtiyari	36 (17.5)	15 (20.3)	51 (18.2)
Lor	27 (13.1)	26 (35.1)	53 (18.9)
Fars	9 (0.04)	2 (0.03)	11 (3.9)
**Education of mothers**			
Illiterate	36 (17.5)	8 (10.8)	44 (15.7)
Primary	101 (49.0)	38 (51.3)	139 (49.6)
Secondary	44 (21.3)	10 (13.5)	66 (23.6)
Diploma	14 (6.7)	10 (13.5)	24 (8.6)
Above diploma	2 (0.9)	5 (6.7)	7 (5.2)
**Education of fathers**			
Illiterate	25 (9.6)	9 (12.16)	34 (12.1)
Primary	76 (3.7)	25 (33.78)	101 (36.1)
Secondary	61 (29.6)	21 (28.4)	103 (36.8)
Diploma	18 (8.7)	3 (4.0)	32 (11.4)
Above diploma	6 (2.9)	4 (5.4)	10 (3.6)
**Occupation of mothers**			
Employee	3 (1.4)	7 (9.4)	10 (3.6)
Self-employed*	4 (1.9)	2 (2.7)	6 (2.1)
Housewife	199 (96.6)	65 (87.8)	264 (94.3)
**Occupation of fathers**			
Employee	21 (10.2)	16 (21.6)	38 (13.6)
Workers	56 (2.7)	18 (24.3)	76 (27.1)
Self-employed*	84 (40.8)	29 (39.2)	113 (40.4)
Unemployed	42 (20.4)	11 (14.9)	53 (18.9)
**Family income per month- IRR**			
Below 1000000	27 (13.1)	2 (2.7)	29 (10.4)
1000000 to 3000000	39 (18.9)	18 (24.3)	57 (20.4)
3000000 to 7000000	78 (37.9)	25 (33.8)	103 (36.8)
7000000 to 10000000	34 (16.5)	17 (22.9)	51 (18.2)
More than 10000000	20 (9.7)	10 (13.5)	30 (10.7)
No response	8 (3.9)	2 (2.7)	10 (3.6)

All data presented as number and percentage, n (%).

U5M = under-five mortality; *Self-employed = agriculture or animal husbandry field.

Most of the subjects were covered by a rural insurance scheme (97%) and above 90% reported to have at least 4 antenatal care (ANC) visits during their pregnancy. Fifty-nine percent of pregnancies occurred below 20 years of age. Cesarean section prevalence was 33%. History of legal abortion was reported by 21.4% of mothers. One-fourth of the families had two or more cases of U5M (Table 3).

**Table 3:** Health-related information about the families with a case of U5M in the previous five years in the rural area of Khuzestan Province.

Health-related information	1 U5M N = 206	>1 U5M N = 74	Total N = 280
**Insurance scheme**			
Yes	195 (94.7)	70 (94.6)	265 (94.6)
No	6 (2.9)	0 (0.0)	6 (2.1)
Missing	19 (9.2)	13 (17.5)	9 (3.2)
**History of antenatal visit**			
Yes	171 (83.0)	53 (71.6)	224 (80.0)
No	16 (7.8)	8 (10.8)	24 (8.5)
Missing	19 (9.2)	13 (17.5)	32 (11.4)
**Maternal age of delivery, for the deceased child**			
14 to 20	110 (53.4)	55 (74.3)	165 (58.9)
20 to 30	73 (35.4)	10 (13.5)	83 (29.6)
30 to 40	22 (10.7	9 (12)	31 (11.1)
40 and above	1 (0.4)	0 (0.0)	1 (0.4)
**Maternal age at first birth**			
**Marriage Age**			
Below 18 years old	86 (41.7)	23 (31.1)	109 (38.9)
18 to 25 years old	98 (47.5)	41 (55.4)	139 (49.6)
25 to 35 years old	17 (8.2)	10 (13.5)	27 (9.6)
Above 35 years old	5 (2.4)	0 (0.0)	5 (1.8)
**History of abortion, legal**			
Yes	42 (20.4)	18 (24.3)	60 (21.4)
No	164 (76.6)	56 (75.7)	220 (78.6)

All data presented as number and percentage, n (%).

U5M = under five mortality.

### The characteristics of the deceased child

Among the recruited children, one-third of the overall cases had Low Birth Weight (LBW). The average birth interval of the deceased child was 1.47 ± 2.28 which represents more than 80% of <3 years interval. Sixty percent of death occurred among children in their first and second birth order. Prematurity and congenital anomaly together with a sum of 46% of all causes of death were the most prevalent with 80% of death accrued during their neonate age and infancy (Table 4).

**Table 4:** Characteristics of the under-five child deceased in the previous five years in rural areas Khuzestan Province.

Characteristics	Number	Valid Percentage
**Sex of the dead child**		
Male	74	53.2
Female	65	46.8
Missing	141	50.04
**Children’s Weight**		
Below 2.5 Kg	39	26.9
Above 2.5 Kg	106	73.1
Missing	135	48.2
**Birth interval**		
Less than one year or 1^st^ child*	184	65.7
Between 1 and 3 years	42	36.8
Between 3 and 5 years	37	32.5
More than 5 years	17	14.9
**Birth order**		
First child	39	27.3
2^nd^ or third child	86	60.1
4^th^, 5^th^ or 6^th^ child	16	11.2
7^th^ or above child	2	1.4
Missing	137	48.9

^*^7 cases were firstborn child.

### Determinants of multiple cases of U5M within a family

The comparison was made between families with one case of U5M and two or more cases of various demographic, socio-economic, and health-related information. Multivariate analysis showed that having >1 under-5 child death was associated with maternal age at delivery (<18 or >35), marriage duration (>9 years), spouse age gap (>5 years), delivery interval (<3 years), ethnicity, and working status of mothers (Table 5). For instance, for mothers being occupied with agricultural or animal husbandry field activity, odds of having more than one under five-child death were 3.9 times larger than not occupied.

**Table 5:** Association between selected socio-demographic and health-related indicators with the presence of more than one child death.

Characteristics	OR (95% CI)	P-value
Non-Central Village	1.01 (0.57 to 1.77)	0.98
Hadi Plan not implemented	0.62 (0.35 to 1.14)	0.12
Delivery Ages < 18 yrs. or >35 yrs.	3.5 (1.19 to 10.22)	0.02
Marriage Duration >9 yrs.	1.85 (1.06 to 3.22)	0.03
Spouse Age Gap >5 yrs.	2.32 (1.20 to 4.50)	0.01
Cesarean Sections	3.849 (1.91 to 5.74)	<0.01
History of abortion, legal	1.255 (0.67 to 2.36)	0.48
Delivery Interval <3 yrs	2.829 (1.22 to 6.58)	0.02
Inadequate ANC visits	1.61 (0.65 to 3.98)	0.29
Non-Arabs Ethnicity	2.58 (1.49 to 4.45)	<0.01
Below Poverty Line	1.60 (0.90 to 2.83)	0.11
Mother working in agriculture or animal husbandry	3.94 (1.41 to 10.98)	<0.01
Father unemployed	1.47 (0.71 to 3.03)	0.3
Mother Education < 6 class	1.21 (0.70 to 2.01)	0.5
Father Education < 6 class	1.14 (0.067 to 1.95)	0.62

Associations are results of multivariate regression and shown in term of Odds Ratio (Exp. Β) and 95% CI. *OR* = odds ratio (calculated as exponentiation of the β coefficient); *95% CI* = 95 percent confidence interval; *Hadi Plan* = also called “Tarh e Hadi” or physical guide plan that facilitated villages through providing extensive physical improvement to the areas; *Delivery Ages* = age of mother at the time of delivery of deceased child; *Delivery Interval* <*3 yrs* = having another delivery within 3-year interval with the deceased child; *ANC visits* = receive at least 4 antenatal care visits; *Poverty Line* = defined as monthly income of <$ 60 USD.

## Discussion

The current study observed that the rural areas in Khuzestan experienced a cumulative incidence of 25 U5M per 1000 live birth during the five years period of 2011 to 2015. The corresponding annual reduction rate was about 3% (from 26 cases per 1000 live birth in 2011 to ≈22 cases per live birth in 2015). However, there was a huge gap between the death rates from place to place (for example 43 in Masjed Soleyman vs. 15 in Dehdez). It has been observed that 97% of families had medical insurance with 90% ANC utilization rate. The literacy rate was close to the national average of 85% reported by UNICEF; however, it was found out that 29% of the participants had monthly income below 60 USD. Prematurity and congenital anomaly were 46% of all the causes of mortality. The study showed important factors associated with having >one U5M, namely, the delivery age of fewer than 18 years or more than 35 years, marriage duration >9 years, spouse age gap >5 years, delivery interval <3 years, cesarean delivery, non-Arab ethnicity, and field activity. For instance, mothers who experience delivery interval <3 years could have 2.83 (95% CI, 1.22 to 5.58) times greater chances to have more than one U5M.

Rarani *et al*.^7^ showed a 15 years (1995–2010) cumulative incidence of 30.4 per 1000 live births in Khuzestan province. The same study indicated almost constant annual reduction rate of 4.7% to 5.0% since 1990^7^. However, we found Khuzestan has a higher U5M than the expected based on the predicted rate of annual decline (expected = 16.72 vs. observed = 22). Simultaneously, national U5M (=19.4) is below the current study observation of 22 and this may show the level of disparity in this indicator in rural Khuzestan as compared to the whole of Iran. Similarly, subnational reports indicating that there are significant differences in mortality rate, for instance, U5M rate for Mazandaran and Zanjan (two of the wealthiest provinces) was 14.6 and 16.4^9^.

Rural areas always suffer from poorer health indicators as compared to urban areas^18,19^. In rural areas, U5M does not only represent general health status but also has an important impact on family planning and birth control. Low U5M creates a sense of security of survival of children for parents to limit the number of their children. The lowest and highest U5M were observed in the neighboring rural areas of Dehdez and Masjed Soleyman, respectively. The primary inhabitant of Dehdez and Masjed Soleyman are Bakhtiari. The rural areas of Masjed Soleyman are impassable, also lack rural settlements due to livestock jobs, and reduced access to health services. However, another speculation for these differences in mortality rate is the fact that Oil Company in Masjed Soleyman attracted other people from other parts and this led to a change in the composition of the population against the health indicator of U5M.

Other studies reported^20,21^ that educational and income status are important factors linked to U5M rate. However, there was no direct observation associated with these factors (that is, education and income) and the odds of having more than one under-five death within a family. A non-significantly higher U5M rate among male as compared to female children was observed, which is mostly related to the biological lower chance of survival of male children and this is a previously well-established factor^12^.

Most of the families with cases of U5M were under types of medical insurance plan and also reported good adherence to antenatal care visit during pregnancy. However, antenatal care visit needs to be adequate in order to avoid the further death of a sibling in childhood (that is, under-five) (Table 5). This may be due to insufficient quality of visits^22^ or non-qualified health house personnel. One-third of the deceased children were LBW (<2500 g), which could contribute to the mortality rate. Early pregnancy (<18 years) and poverty are the main contributors to LBW. The study has found a high prevalence of short birth interval (<3 years) which is associated with U5M^15,23,24^. Also, the quite higher proportion of subjects living below the poverty line (29%) were observed in the present study as compared to the previously reported (13%) in the Iranian rural community^17^. These differences may be due to variation in the qualitative and quantitative methods in the measurements of rural poverty, including social status (that is, social participation, education level of household, credit use), income status (annual household per capita income from agricultural and non-agricultural sources), nutritional status, clothing, housing and social security (that is, use of health and medical insurance)^25^. However, the study did not find a direct association between poverty and the odds of having more than one case of under-five death within a family. We also found being housewives is a protective factor for U5M. We speculated that women who do not have to work are likely wealthier and therefore less likely to have more than one U5M.

In the current study, no association was observed between mother’s education and number of U5M; however, previously, Huda *et al*.^26^ showed the importance of mothers’ age and education in increasing the risks of neonatal, infant, and U5M in Bangladesh. Ethnicity represents many other health determinants like education, birth control, health^26^, behavioral^27,28^, and income level; therefore, more studies are required to assess the link between ethnicity and U5M which can help in designing culturally susceptible and acceptable interventions.

The main limitations of the present study lie in the fact that the main information was secondary with a large number of user-missing values and limited access to the nomad families. Various factors associated with having multiple under-five mortalities have been investigated, but the survey was cross-sectional and this often do not reflect cause and effect relationship and vulnerable to various bias. For instance, the error of the results is when the households with only one U5M may have another in the future. In addition, stillbirths and miscarriages were not considered. Another issue is lack of information on the quality of health care provided. Previous studies showed the poor evidence-based performance of physicians who work in Khuzestan province^22,29^ and this could be a point of concern in a future study. The harsh local environment and widespread poverty in these areas cause an unwillingness of physician to work in these areas unless those who are forced to work as part of their commitment to the government, usually newly graduated and inexperienced graduates.

Due to the demand for achieving MDG4, many countries have taken different measures to reduce U5M. However, all strata of the society have not equally been a beneficiary of this progress. Therefore, the disparity in U5M has increased between and within countries, among rural versus urban areas, and among minority and borderline people. Therefore, special action needs to be taken to address the issue of disparities rather than only targeting national average^12^. Investigating determinants of U5M could help in reducing the disparities.

## Data Availability

The datasets generated during and/or analyzed during the current study are available from the corresponding author on reasonable request.
